# Effects of a nutrition education intervention on nutrition knowledge and attitude among overweight and obese primary schoolchildren: a cluster randomized controlled trial

**DOI:** 10.1186/s12889-025-21671-1

**Published:** 2025-02-04

**Authors:** Rusidah Selamat, Nur Azlina Abdul Aziz, Junidah Raib, Norlida Zulkafly, WNurul Ashikin W. Mohamad, Ainan Nasrina Ismail, Muhammad Yazid Jalaludin, Fuziah Md. Zain, Zahari Ishak, Abqariyah Yahya, Abdul Halim Mokhtar

**Affiliations:** 1 Nutrition Division, Federal Government Administrative Centre, Ministry of Health Malaysia, Level 1, Block E3, Complex E, Putrajaya, 62590 Malaysia; 2 Department of Pediatrics, Faculty of Medicine, Universiti Malaya, Kuala Lumpur, Wilayah Persekutuan Kuala Lumpur 50603 Malaysia; 3 Pediatric Department, Putrajaya Hospital, Precinct 7, Putrajaya, 62250 Malaysia; 4 FOSSLA, UCSI University, Kuala Lumpur, Wilayah Persekutuan Kuala Lumpur 56000 Malaysia; 5 Department of Social and Preventive Medicine, Faculty of Medicine, Universiti Malaya, Kuala Lumpur, Wilayah Persekutuan Kuala Lumpur 50603 Malaysia; 6 Department of Sports Medicine, Faculty of Medicine, Universiti Malaya, Kuala Lumpur, Wilayah Persekutuan Kuala Lumpur 50603 Malaysia; 7 Faculty of Sports and Exercise Science, Universiti Malaya, Kuala Lumpur, Wilayah Persekutuan Kuala Lumpur 50603 Malaysia

**Keywords:** Nutrition education intervention, Childhood obesity, Primary schoolchildren, Cluster randomized controlled trial

## Abstract

**Background:**

School-based obesity interventions are a promising strategy for combating childhood obesity. In this study, we examined the efficacy of the “My Body is Fit and Fabulous at School” (MyBFF@school) program with a nutrition education intervention (NEI) for improving nutrition knowledge and attitude among overweight and obese primary schoolchildren.

**Methods:**

A school-based cluster randomized controlled trial of the MyBFF@school obesity intervention program was conducted among overweight and obese schoolchildren aged 9–11. Out of 1,196 eligible government primary schools in central Peninsular Malaysia, 23 were randomly assigned into seven intervention schools (647 children) and 16 control schools (750 children). A standard nutrition education module was delivered for 24 weeks to the intervention group, whereas children in the control group followed only the currently existing school nutrition education program. The main outcome measures were nutrition knowledge and attitude scores. Changes of nutrition knowledge and attitude scores from follow up until end of 6 months was assessed using the mixed effect model taking into account the cluster effect.

**Results:**

A total of 563 children in the intervention group and 482 in the control group completed the six-month program and were included in the analysis. The overall nutrition knowledge score was significantly higher in the intervention group (adjusted mean difference (AMD): 4.75%, $p=0.028$) after controlling for mean nutrition knowledge score at baseline, gender, location school group (intervention vs control) and ethnicity. There was also a significant improvement in the nutrition knowledge score with AMD among boys (6.02%), urban children (8.07%), and non-Malays (10.4%). In contrast, after controlling for mean nutrition attitude score at baseline, gender, location, school group (intervention vs control) and ethnicity, there was no significant difference in the nutrition attitude scores between the intervention and the control groups in the overall, gender, location and ethnicity.

**Conclusions:**

The MyBFF@school program with an adjunct NEI improves the overall knowledge on nutrition but has no positive effect on the nutrition attitude of younger schoolchildren, necessitating additional improvements.

**Trial registration:**

Clinical trial number: NCT04155255, November 7, 2019 (Retrospective registered). National Medical Research Register: NMRR-13–439-16563. Registered July 23, 2013. The intervention program was approved by the Medical Research and Ethics Committee (MREC), Ministry of Health Malaysia and Educational Planning and Research Division (EPRD), Ministry of Education Malaysia. It was funded by the Ministry of Health Malaysia.

## Background

Over the past two decades, there has been a dramatic increase in childhood obesity in both developed and developing countries. Recent global estimates indicated that about 5.6% or 38.3 million children under the age of five years were overweight in 2019 as compared to about 30.3 million overweight children in 2000 [1]. In addition, among older children, the number of obese 5–19-year-old children rose more than 10 times globally from 11 million in 1975 to 124 million in 2016 [2]. In Malaysia, there is also a higher prevalence of obesity among older children compared to younger children. The National Health and Morbidity Survey (NHMS) 2017 showed an increase in obesity (body mass index [BMI] for age greater than + 2 standard deviations [SDs]) among schoolchildren aged 7–14 from 12.3% in 2012 to 14.8% in 2017 [3]. A higher prevalence of obesity among children aged 7–12 was also reported in urban areas (20.1%) compared to rural areas (13.0%) [4].

Childhood overweight and obesity are generally preventable. Lack of physical activity and unhealthy eating practices are the major contributors to diet-related non-communicable diseases, including obesity. Further, obese children tend to remain obese throughout their adulthood [5, 6]. In Malaysia, the national prevalence of obesity (BMI > 30 kg/m^2^) among adults aged 18 and above has continued to increase from 14.0% in 2006 to 17.7% in 2015 and to 19.7% in 2019 [7–9]. Therefore, a more structured, holistic, and aggressive obesity prevention and control program starting at a young age is urgently required to meet the global targets of no increase in the prevalence of obesity, including childhood obesity, by 2025. Thus, concerted efforts are currently underway by both governmental and non-governmental agencies to combat childhood obesity.

While there have been various small-sacle obesity interventions targeting schoolchildren, a successful larger-scale obesity intervention is required to address this pervasive and growing health threat [10–12]. Several studies have shown that school-based intervention programs combining nutrition education and physical activity are highly effective for obesity prevention and control [13–16]. Obesity in children could help been attenuated by providing appropriate nutrition knowledge and changing the attitude toward healthy eating as early as possible [17, 18].

As children spend more than one-third of their daily life at school, schools could be used as a platform to combat obesity starting at an early age. In Malaysia, there have been various small-scale studies on school-age children but no large-scale school-based obesity intervention [10–12]. This program, “My Body is Fit and Fabulous at School” (MyBFF@school), is a school-based obesity intervention that consists of multiple components: physical activity in the form of small-sided games (SSGs), nutrition education intervention (NEI), and psychological empowerment. The primary aim of the MyBFF@school study is to reduce overweight and obesity in primary schoolchildren, whereas the aim of the current study is to examine the effects of the NEI component on nutrition knowledge and attitude among overweight and obese primary schoolchildren participating in MyBFF@school compared to overweight and obese children following the currently existing school nutrition program.

## Methods

**Fig. 1:**
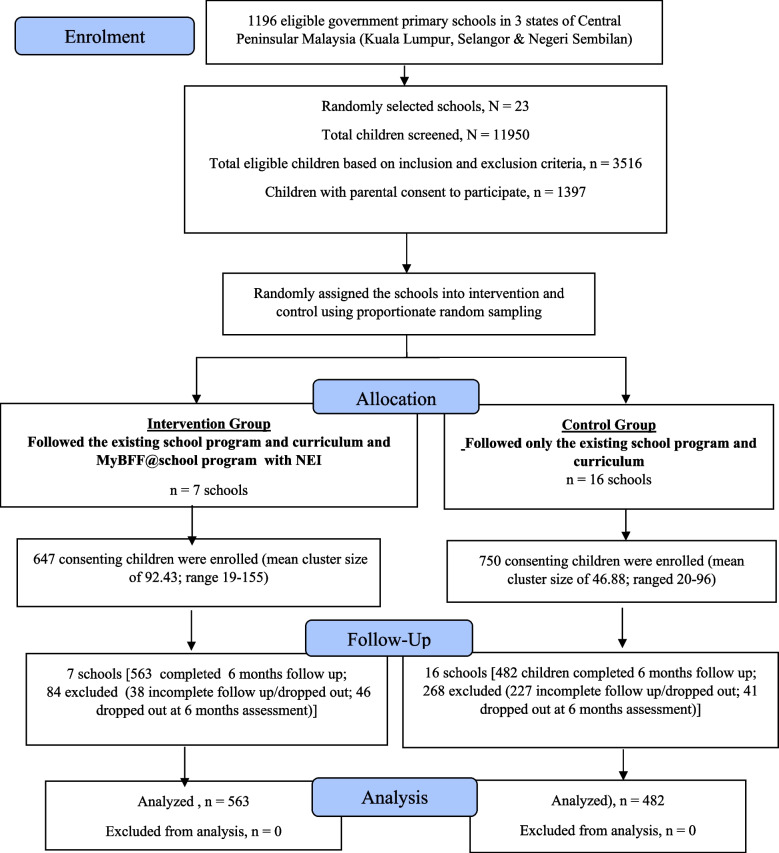
CONSORT diagram for nutrition component in MyBFF@school

The data collected for this study were part of the My Body is Fit and Fabulous at School (MyBFF@school) obesity intervention program, a cluster randomized control trial. The detailed methodology for the MyBFF@school intervention including the sampling, sample size calculation, recruitment and participation eligibility is described by Mokhtar et al. [19]. The target population of the MyBFF@school intervention were school children aged 9–11 years old attending government primary schools in the three states in the central region of Peninsular Malaysia namely Kuala Lumpur, Selangor and Negeri Sembilan (Fig. 1). These schools were considered as urban or rural based on the current classification used by the Ministry of Education adopted from the Department of Statistics Malaysia (DOSM) 2010 where the same definition was used for the National Census by DOSM [20]. Out of 1,196 eligible government primary schools, 23 were randomly selected and assigned into the intervention (7 schools; 647 school children) and control (16 schools; 750 school children) taking into consideration the school type and locality as shown in Fig. 1. In brief, the minimum sample size needed for the study was 402 subjects per arm with mean difference of 0.35 in the percentage of body fat and 80% power. With the assumption of intraclass correlation coefficient (ICC) of 0.01 and 50% attrition rate, the sample size required was 1200 subjects. In our study, a desired sample size was reached with 23 schools. Parental or guardian written informed consent was obtained via the distribution of the written consent form to the school administrator in charge of helping in the coordination of MyBFF@school intervention.

Schools that were involved in other overweight and obesity intervention programs were excluded from the study. Special schools, such as boarding schools, private schools, and international schools, were also excluded. While the control and intervention schools were considered independent samples, certain factors were beyond our control, such as shared friends and shared experiences. Allocation concealment was not possible since the list of the selected schools must be submitted to the Ministry of Education Malaysia prior to the study as part of the overall approval process or procedures to conduct the school based study. Similarly, the blinding of the subjects was not possible since the subjects who participated in the MyBFF@school program were from the same school with an open nature of intervention such as SSG and nutrition.

The inclusion criteria for selecting the children were overweight and obese children with BMI for age z-score of more than + 1SD based on WHO 2007 Growth Reference [21] with age between 9–11 years old, in standard 3, 4 and 5 for primary schools. The BMI for age z-score category used in our study was overweight (> + 1SD to ≤  + 2SD); obese (> + 2SD to ≤ 3SD) and morbidly obese (> + 3SD. In our study, only children age 9–11 years old were chosen although the children attended the primary school ranged from 7 to 12 years old. Younger children age 7 and 8 years old were excluded since there were too young to respond to questionnaire in the MyBFF component which include nutrition and psychology while older children age 12 year old (Year 6) were excluded since these children involved in major National Examination. The children exclusion criteria were (1) BMI for age below + 1 SD; (2) physical or mental disability; (3) medical conditions preventing participation in moderate-to-vigorous physical activity; (4) comorbidities that may interfere with the study (e.g., type 2 diabetes mellitus, hypertension, nephritic syndrome, epilepsy, congenital heart disease, and skeletal anomalies); and (5) current use of steroids, antiepileptics, or methylphenidate. This study was approved by the Medical Research and Ethics Committee, Ministry of Health Malaysia (NMRR no. 13–439-16,563).

### MyBFF@school intervention with NEI

The MyBFF@school intervention incorporated NEI, physical activity in the form of SSGs, and a psychological empowerment component.The nutrition component in the form of NEI was designed as a nutrition education module (NEM) specifically to address childhood obesity. The NEM was developed and delivered using interactive methods adapted from the Malaysian Childhood Obesity Treatment Trial (MASCOT) [22]. There were five main topics, each divided into several subtopics (refer to Table 1). These subtopics covered “challenges in body weight loss and management,” “know more about your body weight and whether you are at risk,” “how to eat a well-balanced meal,” “how to increase fruit and vegetable intake,” “how to increase plain water consumption,” “how to reduce snacking,” “how to have an appropriate breakfast,” “how to prepare a healthy meal,” “smart shopping,” and “tips when eating outside home.”

**Table 1:** Module for NEI for primary schoolchildren

Topic	Subtopic	Objective	Activity included
**Topic 1:** Wake-Up Call/Time to Act	Unit 1: Are You at Risk?	To create awareness among the target groups (parents, teachers, and students) on unhealthy food intake	Class teaching
	Unit 2: Challenges in Body Weight Loss and Management	To expose the target groups (parents, canteen operators, teachers, and students) to challenges of weight loss management	
	Unit 3: Time to Act	To provide exposure to the parents on nutrition intervention sessions that must be taken by the students in the MyBFF@school program	
**Topic 2:** My Body Weight/Know My Body Weight	Unit 1: My Body Weight/Know My Body Weight	1. To perform anthropometric measurements (height and weight) 2. To calculate the BMI and plot the data on growth charts 3. To interpret the results of BMI for age	Practical session on weight and height measurement, calculation and interpretation of BMI using growth charts
**Topic 3:** Eat Well, Be Well	Unit 1: A Balancing Act/Count to Be Fit	1. To know the concept of energy balance 2. To know the sources and total energy intake 3. To know the total energy consumption	Interactive game on getting to know the food pyramid and physical activity pyramid
	Unit 2: Fill In My Plate/Healthy Eating Plan	1. To understand and apply the concept of the Malaysian Food Pyramid/Healthy Plate 2. To understand menu planning	Practical session on how to plan meals with the Malaysian Healthy Plate
	Unit 3: Awesome Fruits and Veggies	1. To know the importance of daily fruit and 2. vegetable consumption 3. To know the ways of increasing the intake of fruits and vegetables 4. To understand and apply the number of recommended servings	Fruit serving size drawing
	Unit 4: Plain Water My Best Friend	To know the importance of drinking plain water	Interactive session to determine the amount of sugar content in beverages
	Unit 5: Less Salt and Fat/Snack Attack	To guide the students on how to choose a healthy snack including foods with less salt and fat	Interactive session to guess the amount of salt/sodium content in food
**Topic 4:** Make a Better Life	Unit 1: Breakfast Power	1. To know the importance of breakfast 2. To know the benefits of breakfast in learning 3. To know about healthy breakfast options	Interactive session with the students on a sample of healthy breakfast
**Topic 5:** MyBFF Smart Shopping, Preparing Meal Together and Eating Out	Unit 1: Smart Shopping	1. To provide guidance to the students on how to read and understand food labels and how to choose food wisely 2. To provide guidance to the students on how to prepare healthy foods and practice healthy eating 3. To provide guidance on how to make healthy food choices when eating out	Classroom teaching
	Unit 2: Let’s Cook/Prepare Meal Together	To teach children how to prepare healthy food	Preparing healthy balanced meals (e.g., sandwiches)
	Unit 3: Eating Out: Fast Food/Food Outlet/School Canteen/Hawkers	To provide information on how to make healthy choices when eating out	

A different dedicated trained staff with nutrition degree was stationed in the seven intervention schools to conduct NEI using NEM during the co-curriculum activities after the school hours in each respective school for 24 weeks. These staffs were employed under the MyBFF@school research project and had undergone a centralized training by the research team on how to use the NEI module. There were dedicated field supervisors which consisted of the research team to monitor and assure the adherence to the NEM. Each NEI session was conducted once every 2 weeks for 45–60 min per session for a total of 12 contact hours, at alternate week with psychology component. As noted in Table 1, parents were involved in only the three units under Topic 1. Lectures and nutrition education sessions with interactive discussions were conducted using slideshows and practical sessions, such as on how to determine the amount of sugar in beverages, preparing healthy meals, learning the serving sizes of fruits and vegetables, and meal planning using the Malaysian Healthy Plate and Food Pyramid.

SSG sessions were conducted twice a week for 30 min, and each SSG involved four to seven participants per team. The SSGs included a foot game, a hand game, and other games requiring the participants to move and run around the pitch. The psychological component included motivational talks and games with themes of friendship, self-esteem, assertiveness, positive thinking for a healthy life, and stress management conducted for 30–45 min per session on alternating weeks with the NEI.

Apart from the MyBFF@school intervention, the intervention schools were also following the standard existing school programs and curriculum whereas the control group were only following the existing school program and curriculum. In Malaysia, the normal health curriculum for primary schoolchildren runs for six years from the age of seven until the age of 12. It is incorporated into the school curriculum as part of health education and is mandatory in all government schools. Apart from nutrition, other health components covered in this curriculum are mental health, personal hygiene, reproductive health, and selected diseases. This health education subject is taught over 35 sessions (30 min each) per year. However, the nutrition component is only allocated five sessions (15%), equivalent to 150 min per year. In addition, no hands-on or interactive session is included in the nutrition component.

### Anthropometric and body composition measurements

Body height was measured without shoes and socks to the nearest 0.1 cm using a calibrated stadiometer (Seca 217; Seca, Hamburg, Germany). Body weight, body fat mass, skeletal muscle mass (SMM), and percentage body fat (PBF) were measured using a body impedance analyzer (InBody 720; InBody, Seoul, Korea).

### Baseline and post-intervention assessments of nutrition knowledge and attitude

A field-tested questionnaire was administered at baseline (pre) and after six months (post) to both the intervention and control groups to assess nutrition knowledge and attitude. This questionnaire was developed by the MyBFF@school NEI component team that comprises nutritionists and health education officers from academia and the Ministry of Health Malaysia. The 10-question general nutrition knowledge section included items on how breakfast can reduce overeating, daily vegetable servings (two), plain daily water intake (six to eight glasses), and the dangers of sugared carbonated beverages. Questions on fast food, cooking methods, and BMI measurement were also included. A score of 1 was given to the correct answer, whereas wrong answers (or no answer at all) were given a score of 0. The total score for every respondent was calculated and converted into a percentage for tertile comparison. Cronbach’s alpha coefficient for the 10-item nutrition knowledge scale was 0.673. The 15-question key nutrition attitude component also included items on the intake of fruits and vegetables, breakfast, plain water, fried food, sweet food, carbonated drinks, and fast food. The attitude items were scored on a five-point Likert scale ranging from “strongly disagree” (1 point) to “strongly agree” (5 points). An intermediate (neutral) option was allocated 3 points. Cronbach’s alpha coefficient for the 15-item nutrition attitude scale was 0.679. The total scores for nutrition attitude items were also converted into a percentage.

### Statistical analysis

REDCap electronic data capture tools were used to manage all data. A web-based application was designed to support data capture for research studies [23]. Descriptive statistics were used to describe the baseline data. Categorical variables were compared between groups using the chi-squared test. Scores within groups (“post” versus “pre”) without controlling the covariates was done using a paired samples *t*-test. Changes of nutrition knowledge and attitude scores from follow up until end of 6 months was assessed using the mixed effect model taking into account the cluster effect. All data analyses were conducted using SPSS Statistics (Version 24; IBM Corp., Armonk, NY, USA) except for the intraclass correlation coefficient (ICC) and mixed effect model which were analysed using STATA Version 14. A $p$-value of < 0.05 (two-tailed) was considered significant for all tests.

## Results

### Baseline characteristics

The study flow is shown schematically in the CONSORT diagram in Fig. 1. Out of the 1,397 participants recruited, 352 (25.2%) were excluded from the analyses because of dropping out or incomplete data. The remaining 1,045 respondents included 563 from the intervention group and 482 from the control group. The age distribution of the study population was between 9 and 11 years (mean age: 9.94 years), and the majority were Malays (83.0%). More respondents were boys (52.6%) and living in urban areas (59.2%). There were no significant differences in the gender ratio, urban/rural distribution, and age distribution between the groups at baseline. There were also no significant differences in the anthropometric indicators between the groups at baseline. There was a significant difference in the ethnic distribution between the groups with more Malays were in the control group whereas more non-Malays were in the intervention group (Table 2).

**Table 2:** Baseline characteristics of the study schoolchildren

**Characteristics**	**Intervention group**	**Control group**	χ^2^	$p$-value
	($n=563$)	($n=482$)		
**Gender, ** ${\varvec{n}}$** (%)**^**a**^				
Boys	297 (52.8)	253 (52.5)	0.007	0.932
Girls	266 (47.2)	229 (47.5)		
**Location, ** ${\varvec{n}}$** (%)**^**a**^				
Urban	324 (57.5)	295 (61.2)	1.436	0.231
Rural	239 (42.5)	187 (38.8)		
**Age group, ** ${\varvec{n}}$** (%)**^**a**^				
9 years old	193 (34.3)	157 (32.6)	0.476	0.788
10 years old	218 (38.7)	187 (38.8)		
11 years old	152 (27.0)	138 (28.6)		
**Ethnicity, ** ${\varvec{n}}$** (%)**^a***^				
Malay	433 (76.9)	434 (90.0)	31.688	< 0.001***
Non-Malay^c^	130 (23.1)	48 (10.0)		
**School category, ** ${\varvec{n}}$** (%)**^a***^				
National school (Malay)	435 (77.3)	440 (91.3)	37.481	< 0.001***
National school (Chinese and Tamil)	128 (22.7)	42 (8.7)		
**BMI category, ** ${\varvec{n}}$** (%)**^**a**^				
Overweight	222 (39.4)	179 (37.1)	2.400	0.301
Obese	243 (43.2)	201 (41.7)		
Morbidly obese	98 (17.4)	102 (21.2)		
**Anthropometric status, mean (SD)**^**b**^				
Body weight (kg)	46.14 (10.64)	46.91 (10.88)		0.250
Body height (cm)	139.12 (7.78)	138.92 (7.46)		0.670
BMI for age$z$-score	2.30 (0.84)	2.37 (0.90)		0.160
Skeletal muscle mass (kg)	14.68 (2.93)	14.73 (2.90)		0.762
Body fat mass (kg)	17.85 (6.75)	18.68 (6.88)		0.050
Percentage body fat (%)	37.73 (6.52)	38.75 (6.51)		0.070

***p<0.001

^a^Chi-square Test

^b^Independent T-Test

^c^The ethnicity for the Non-Malay has been regrouped which consisted of Chinese, Indians and others

### Nutrition knowledge and attitude scores at baseline and after nutrition education in the highest tertile groups

At baseline, about 40% of the schoolchildren were at the highest tertile of nutrition knowledge scores (70–100%) in both the intervention and the control groups (Table 3). There was a reduction in the nutrition knowledge scores within the highest tertile after six months for both the intervention and the control groups. A similar pattern was observed for boys, girls, Malays, and non-Malays. There were no significant differences in the nutrition attitude scores relative to the baseline for the highest tertile of boys, Malays, and the entire intervention group.

**Table 3:** Nutrition KA score at baseline and after 6 months between the intervention and control group

**Parameter based on tertile**	**At Baseline, n (%)**			**After 6 month, n (%)**		***p*-value for** **Chi – square Test**
	**Intervention (*n* = 563)**	**Control (*n* = 482)**	***p*-value for Chi-square Tests**	**Intervention (*n* = 563)**	**Control** **(*n* = 482)**	
**KNOWLEDGE**						
** Overall**						
Lowest (≤ 50%)	192 (34.1)	198 (41.1)		200 (35.5)	198 (41.1)	
Middle (> 50- < 70%)	134 (23.8)	82 (17.0)	0.010*	208 (36.9)	176 (36.5)	0 .090
Highest (70–100%)	237 (42.1)	202 (41.9)		155 (27.5)	108 (22.4)	
** Gender**						
** Boys**	*n* = 297	*n* = 253		*n* = 297	*n* = 253	
Lowest (≤ 50%)	99 (33.3)	106 (41.9)		115 (38.7)	120 (47.4)	
Middle (> 50- < 70%)	70 (23.6)	42 (16.6)	0.049*	105 (35.4)	80 (31.6)	0 .109
Highest (70–100%)	128 (43.1)	105 (41.5)		77 (25.9)	53 (20.9)	
** Girls**	*n* = 266	*n* = 229		*n* = 266	*n* = 229	
Lowest (≤ 50%)	93 (35.0)	92 (40.2)		85 (32.0)	78 (34.1)	
Middle (> 50- < 70%)	64 (24.1)	40 (17.5)	0.174	103 (38.7)	96 (41.9)	0 .413
Highest (70–100%)	109 (41.0)	97 (42.4)		78 (29.3)	55 (24.0)	
** Ethnicity**						
** Malay**	*n* = 433	*n* = 434		*n* = 433	*n* = 434	
Lowest (≤ 50%)	166 (38.3)	172 (39.6)	0 .007**	143(33.0)	168 (38.7)	0.107
Middle (> 50- < 70%)	110 (25.4)	74 (17.1)		161 (37.2)	161 (37.1)	
Highest (70–100%)	157 (36.6)	188 (43.3)		129 (29.8)	105 (24.2)	
** Non-Malay**	*n* = 130	*n* = 48		*n* = 130	*n* = 48	
Lowest (≤ 50%)	26 (20.0)	26 (54.2)	< 0.001***	57 (43.8)	30 (62.5)	0.033*
Middle (> 50- < 70%)	24(18.5)	8 (16.7)		47 (36.2)	15 (31.2)	
Highest (70–100%)	80 (61.5)	14 (29.2)		26 (20.0)	3(6.2)	
** Location**						
** Urban**	*n* = 324	*n* = 295		*n* = 324	*n* = 295	
Lowest (≤ 50%)	73 (22.5)	130 (44.1)	< 0 .001***	111 (34.3)	115 (39.0)	0.029*
Middle (> 50- < 70%)	80 (24.7)	47 (15.9)		112 (34.6)	116 (39.3)	
Highest (70–100%)	171 (52.8)	118 (40)		101 (31.2)	64 (21.7)	
** Rural**	*n* = 239	*n* = 187		*n* = 239	*n* = 187	
Lowest (≤ 50%)	119 (49.8)	68 (36.4)	< 0 .001***	89 (37.2)	83 (44.4)	0 .198
Middle (> 50- < 70%)	54 (22.6)	35 (18.7)		96 (40.2)	60 (38.5)	
Highest (70–100%)	66 (27.6)	84 (44.9)		54 (22.6)	44 (23.5)	
**ATTITUDE**						
** Overall**	*n* = 563	*n* = 482		*n* = 563	*n* = 482	
Lowest (≤ 50%)	207 (36.8)	154 (32.0)		174 (30.9)	150 (31.1)	
Middle (> 50- < 70%)	181 (32.1)	172 (35.7)	0.242	224 (39.8)	156 (32.4)	0.018*
Highest (70–100%)	175 (31.1)	156 (32.4)		165 (29.3)	176 (36.5)	
** Gender**						
** Boys**	*n* = 297	*n* = 253		*n* = 297	*n* = 253	
Lowest (≤ 50%)	115 (36.4)	92 (38.7)		106 (35.7)	89 (35.2)	
Middle (> 50- < 70%)	97 (32.7)	90 (35.6)	0.757	118 (39.7)	84 (33.2)	0.133
Highest (70–100%)	85 (28.6)	71 (28.1)		73 (24.6)	80 (31.6)	
** Girls**	*n* = 266	*n* = 229		*n* = 266	*n* = 229	
Lowest (≤ 50%)	92 (34.6)	62 (27.1)		68 (25.6)	61 (26.6)	
Middle (> 50- < 70%)	84 (31.6)	82 (35.8)	0.196	106 (39.8)	72 (31.4)	0.121
Highest (70–100%)	90 (33.8)	85 (37.1)		92 (34.6)	96 (41.9)	
** Ethnicity**						
** Malay**	*n* = 433	*n* = 434		*n* = 433	*n* = 434	
Lowest (≤ 50%)	125 (28.9)	131 (30.2)	0.878	123 (28.4)	128 (29.5)	0.042*
Middle (> 50- < 70%)	153 (35.3)	154 (35.5)		174 (40.2)	141 (32.5)	
Highest (70–100%)	155 (35.8)	149 (34.3)		136 (31.4)	165 (38.0)	
** Non-Malay**	*n* = 130	*n* = 48		*n* = 130	*n* = 48	
Lowest (≤ 50%)	82 (63.1)	23 (47.9)	0.089	51 (39.2)	22 (45.8)	0 .645
Middle (> 50- < 70%)	28 (21.5)	18 (37.5)		50 (38.5)	15 (31.2)	
Highest (70–100%)	20 (15.4)	7 (14.6)		29 (22.3)	11 (22.9)	
** Location**						
** Urban**	*n* = 324	*n* = 295		*n* = 324	*n* = 295	
Lowest (≤ 50%)	134(41.4)	95(32.2)	0.062	102(31.5)	86(29.2)	0.231
Middle (> 50- < 70%)	95(29.3)	101(34.2)		119(36.7)	96(32.5)	
Highest (70–100%)	95(29.3)	99 (33.6)		103(31.8)	113(38.3)	
** Rural**	*n* = 239	*n* = 187		*n* = 239	*n* = 187	
Lowest (≤ 50%)	73(30.5)	59(31.5)	0.803	72(30.1)	64(34.2)	0.039*
Middle (> 50- < 70%)	86(36.0)	71(38.0)		105(43.9)	60(32.1)	
Highest (70–100%)	80(33.5)	57(30.5)		62(25.9)	63(33.7)	

^*^p < 0.05

^**^p < 0.01

^***^p < 0.001

### Effects of the NEI on nutrition knowledge and attitude scores

The within group analysis without adjusting or controlling the mean nutrition knowledge or attitude score at the baseline, gender, location and ethinicity showed a significant increase in the nutrition knowledge score in the intervention group (Table 4). However, there was a significant reduction of nutrition knowledge score in the control group and also a significant reduction of nutrition attitude after 6 months in both the intervention and control group (Table 4). In contrast to nutrition knowledge, nutrition attitude score decreased from the baseline among rural and Malay children of the intervention group (Table 4), whereas there was a significant increase in attitude among non-Malay children of the intervention group.

**Table 4:** Mean KA scores at baseline and after six months for the intervention and control groups

**Parameters**	**Intervention group**^**a**^** (** ${\varvec{n}}=563$**)** **Mean ± SD**		${\varvec{p}}$**-value**	**Control group**^**a**^** (** ${\varvec{n}}=482$**)** **Mean ± SD**		${\varvec{p}}$**-value**
	**Baseline**	**After 6 months**		**Baseline**	**After 6 months**	
**NUTRITION KNOWLEDGE**						
**Overall**	60.15 ± 16.85	60.95 ± 18.90	< 0.001	57.82 ± 20.70	56.82 ± 20.70	< 0.001***
**Gender**						
Boys	60.67 ± 17.11	59.56 ± 19.28	0.424	57.15 ± 20.96	54.34 ± 21.65	0.065
Girls	59.58 ± 16.58	62.51 ± 18.36	0.025*	58.55 ± 17.54	59.56 ± 19.28	0.475
**Location**						
Urban	64.38 ± 15.41	61.69 ± 19.33	0.035*	56.81 ± 20.16	58.27 ± 19.76	0.257
Rural	54.43 ± 17.06	59.95 ± 18.29	< 0.001***	59.41 ± 18.08	54.54 ± 21.97	0.005**
**Ethnicity**						
Malay	58.29 ± 16.16	62.49 ± 18.41	< 0.001***	58.24 ± 19.44	57.83 ± 20.75	0.700
Non-Malay	66.38 ± 17.65	55.84 ± 19.67	< 0.001***	53.95 ± 18.87	47.70 ± 18.01	0.098
**NUTRITION**						
**ATTITUDE**						
**Overall**	67.27 ± 10.42	66.70 ± 9.41	< 0.001***	68.02 ± 10.37	67.41 ± 10.17	< 0.001***
**Gender**						
Boys	66.52 ± 10.15	65.67 ± 9.09	0.143	67.01 ± 10.89	66.08 ± 9.76	0.214
Girls	68.10 ± 10.67	67.85 ± 9.63	0.720	69.14 ± 9.67	68.87 ± 10.43	0.687
**Location**						
Urban	66.67 ± 10.80	66.97 ± 9.73	0.617	68.14 ± 10.58	67.68 ± 9.97	0.471
Rural	68.07 ± 9.84	66.33 ± 8.96	0.007**	67.84 ± 10.08	66.98 ± 10.49	0.295
**Ethnicity**						
Malay	68.75 ± 10.34	67.37 ± 9.15	0.004**	68.61 ± 10.21	67.83 ± 10.09	0.141
Non-Malay	62.32 ± 9.10	64.47 ± 9.92	0.036*	62.72 ± 10.47	63.61 ± 10.18	0.578

^a^Paired T-Test

^*^ $$p<0.05$$

^**^ $$p<0.01$$

^***^ $$p<0.001$$

The effects of NEI had shown that an overall nutrition knowledge score was significantly higher in the intervention group [adjusted mean difference (AMD): 4.75%, $$p=0.028$$] after controlling for mean nutrition knowledge score at baseline, gender, location, school group (intervention vs control) and ethnicity (Table 5). There was also a significantly higher nutrition knowledge score in the intervention group for boys and those children in the urban areas with AMD of 6.02%, $$p=0.020$$ and 8.07%, $$p=0.008$$ respectively. Although girls and children in rural areas in the intervention group demonstrated an increase in the nutrition knowledge score, the AMD was however not significant. As shown in Table 5, there was also a significant positive mean difference of nutrition knowledge score among non-Malays in the intervention and control groups (AMD: 10.40%, $$p=0.017)$$ after controlling for mean nutrition knowledge score at baseline, gender, location, school group (intervention vs control) and ethnicity while there was no significant difference among Malays. As for the nutrition attitude score, after controlling for mean nutrition attitude score at baseline, gender, location school group (intervention vs control) and ethnicity, there was no significant difference in the AMD of nutrition attitude scores between the intervention and the control groups in the overall, gender, location and ethnicity (Table 5).

**Table 5:** Unadjusted and adjusted nutrition knowledge and attitude score

	**DESCRIPTIVE (baseline)**				**MODEL**^**d**^				
	**Intervention**		**Control**		**Crude model**^**a**^	**Adjusted model**^**b**^			
	**N**	**Mean (SD)**	**N**	**Mean (SD)**	**Comparative statistic**^c^	**Comparative statistic**^c^	**95% CI**	***p***	**ICC**
**Nutrition knowledge score (%)**									
**Overall**	563	60.16 (16.86)	482	57.82 (19.41)	3.58	4.76	0.52, 8.99	0.03*	0.049
**Gender**									
Boys	297	60.67 (17.11)	253	57.15 (20.96)	5.17	6.02	0.93, 11.11	0.02*	0.055
Girls	266	59.59 (16.58)	229	58.56 (17.55)	2.24	2.92	−1.52, 7.37	0.20	0.051
**Location**									
Urban	324	64.38 (15.42)	295	56.81 (20.17)	5.78	8.07	2.14, 14.01	0.008**	0.056
Rural	239	54.44 (17.06)	187	59.41 (18.09)	0.81	0.90	−3.75, 5.55	0.71	0.020
**Ethnicity**									
Malay	433	58.29 (16.17)	434	58.25 (19.44)	1.76	2.30	−1.75, 6.35	0.27	0.033
Non-Malay	130	66.38 (17.65)	48	53.96 (18.88)	9.96	10.41	1.86, 18.95	0.02*	0.071
**Nutrition attitude score (%)**									
**Overall**	563	67.27 (10.42)	482	68.03 (10.38)	−0.82	0.01	−1.35, 1.37	0.99	0.011
**Gender**									
Boys	297	66.53 (10.15)	253	67.01 (10.90)	−0.37	0.36	−1.72, 2.44	0.73	0.031
Girls	266	68.10 (10.67)	229	69.15 (9.67)	−1.01	−0.45	−1.87, 0.98	0.54	0.004
**Location**									
Urban	324	66.67 (10.81)	295	68.14 (10.58)	−2.01	0.18	−1.38, 1.74	0.82	0.006**
Rural	239	68.08 (9.84)	187	67.84 (10.08)	0 .73	0 .64	−1.79, 3.06	0.61	0.020
**Ethnicity**									
Malay	433	68.75 (10.35)	434	68.61 (10.21)	−0.12	−0.03	−1.24, 1.18	0.96	0.005**
Non-Malay	130	62.33 (9.10)	48	62.72 (10.47)	3.53	3.67	−1.26, 8.61	0.14	0.093

ICC – intra cluster (intra-school) correlation coefficient

^*^ $$p<0.05$$

^**^ $$p<0.01$$

^***^ $$p<0.001$$

^a^Adjusted for school mean score (nutrition knowledge and attitude) at baseline

^b^Adjusted for school mean score at baseline, gender, location, ethnicity and school group (intervention vs control)

^c^Mean difference (intervention vs control)

^d^Results from mixed effects models

## Discussion

This NEI incorporated into the MyBFF@school intervention program significantly improved nutrition knowledge (AMD: 4.75%, $p=0.028$) after controlling for mean nutrition knowledge score at baseline, gender, location school group (intervention vs control) and ethnicity, consistent with previous studies demonstrating that school-based NEIs are useful for lifestyle and behavioral modifications [24, 25]. However, there was no significant mean difference on the nutrition attitude scores after controlling for the nutrition attitude score at baseline, school group (intervention vs control) and ethnicity. This could be attributed to a shorter 12 h program length distributed over 24 weeks, as a previous study showed that sufficient improvement in nutrition knowledge and behavioral changes require at least 50 h [26]. Unfortunately, limited time can be allocated to the NEI; therefore, increasing the number of hours would necessarily increase the total program duration. Nonetheless, this brief program did improve nutrition knowledge of the schoolchildren.

While there was an overall mean nutrition knowledge score increased in the intervention group, there was perhaps because of a reduction in the control group, suggesting that the final group difference in the knowledge score is probably an artifact of higher dropout rate in the control group. Control students with greater knowledge may not be interested or motivated to complete the program, resulting in a lower nutrition attitude score. There were also gender differences in these scores. Boys achieved a significantly higher nutrition knowledge score in the intervention group with AMD of 6.02%, $p=0.020$ as compared to those in the control group, whereas there was no significant difference between girls in the intervention and control groups. In contrast, in a study in Seoul, Korea, it was reported that girls achieved a greater increase in nutrition knowledge compared to boys [27].

As for the nutrition attitude score, after controlling for mean nutrition attitude score at baseline, gender, location school group (intervention vs control) and ethnicity, although there was a increase in AMD in the overall, boys, urban, rural and non-Malay as well as a reduction of the AMD among girls and Malays, these AMDs were however not significant. The possible factors that might contribute to no significant increase in the nutrition attitude could possibly be attributed by several factors such as lacking of parental support at home apart from these children have not yet developed the diet related diseases or encountered the negative impact of obesity such as diabetes and hypertension. Therefore, this would subsequently lead them to not really appreciate the importance of having or achieving a normal weight. Alternatively, Choi et al. reported slightly higher or better nutrition attitude in girls (7.59 points) compared to boys (7.31 points) [18]. These differences suggest that girls show greater interest than boys in activities like food preparation and nutrition despite having lower nutrition knowledge than boys. In the Malaysian context and culture, there is however still some gender differences whereby girls are more interested in more feminine types of activities such as food and nutrition even from the young age.

The study employed several techniques to ensure the effectiveness of the intervention, such as displaying the caloric contents of foods and beverages sold in the school canteen and setting up nutrition-related education displays within the school compound to help reinforce lessons. Although dedicated trained staff were assigned to deliver the NEI in each intervention school, the nutrition attitude score at six months was actually lower than at the baseline in both the intervention and the control groups. This may be attributed to the extensive after-school activities attended by these children, including the other MyBFF@school components (psychological empowerment and SSGs). Thus, additional factors, such as changes in the physical environment and better accessibility to healthy foods, may be required to promote positive behavioral and nutritional outcomes in children. In addition, more extensive and hands-on nutrition education may be needed to improve or strengthen their understanding and attitude.

There are several limitations to this study. First, there was no direct and persistent parental involvement throughout the study period aside from a special briefing on what was expected from the obesity intervention program at the beginning of the program. Because of the logistic and academic pressure, especially in urban areas, fewer schoolchildren participated at six months. In addition, participation in the program was voluntary and the control group followed already existing school programs and curriculum with no additional component, which may have further exacerbated the dropout rate compared to the intervention group. In addition, as the NEI was conducted after-school hours, some of the children were not able to be fully committed, which subsequently affected the overall effectiveness of the NEI. Therefore, to further strengthen the implementation of obesity intervention program among school children, it requires a multi-pronged strategy and commitment from all the relevant key players including the parents and the children themselves. To certain extent, continuous efforts should be made to empower these school children to be responsible for their own health from the very young age.

## Conclusion

MyBFF@school plus NEIs appears to improve the overall nutrition knowledge of overweight and obese schoolchildren, which is essential for managing childhood obesity. Evidence suggests that appropriately tailored NEIs are likely to reduce the prevalence of childhood obesity [28, 29]. These findings highlight the need for NEIs in programs for overweight and obese schoolchildren. However, MyBFF@school plus NEIs did not significantly improve the nutrition attitude of overweight and obese schoolchildren which remains a challenge for the healthcare providers. In addition to strong multidisciplinary teams of healthcare providers, the commitment and support of parents and schoolteachers are crucial for ensuring the inculcation of positive attitude on nutrition. Thus, contributions and participation of parents may be required to optimize program success. Nonetheless, given the heterogenous participant population, this NEI may be broadly applicable and the study results may be generalizable to children of different ethnic and socioeconomic backgrounds.

## Data Availability

All relevant data are within the paper.

## Abbreviations

AMD: Adjusted Mean Difference
SSG: Small Sided Games
ICC: Intra Class/ Cluster Correlation Coefficient
IPH: Institute of Public Health
NEI: Nutrition Education Intervention
NEM: Nutrition Education Module
NHMS: National Health and Morbidity Survey
MyBFF@school: My Body is Fit and Fabulous at school
SD: Standard Deviation
